# Effect of Drying and Microwave-Assisted Extraction Parameters on *Lippia adoensis* Variety Koseret Essential Oil Yield

**DOI:** 10.17113/ftb.62.04.24.8611

**Published:** 2024-12

**Authors:** Bethlehem Worku, Nurelegne Shibeshi, Jong-Bang Eun, Youngmo Yoon

**Affiliations:** 1 School of Chemical and Bioengineering, Addis Ababa Institute of Technology, Addis Ababa University, King George 04, 1000 Addis Ababa, Ethiopia; 2 Department of Integrative Food, Bioscience and Biotechnology, Graduate School of Chonnam National University, 77 Yongbong-ro, Buk-gu, 61186 Gwangju, South Korea; 3 Hanbit Flavor and Fragrance Co. Ltd., 88 Sinwon-ro, Youngtong-gu, 101-1511 Gyeonggi-do, South Korea

**Keywords:** *Lippia adoensis* variety koseret essential oil, Box Behnken design, room and oven drying of leaves, microwave-assisted extraction

## Abstract

**Research background:**

Green extraction technologies, such as microwave-assisted extraction, have been used to replace conventional methods of isolating essential oils from plants. In this study, the essential oil was extracted from the *Lippia adoensis* variety koseret using the advanced method of microwave-assisted hydrodistillation. The main objective was to investigate the effect of irradiation time, microwave power and particle size on the yield and chemical composition of the essential oil extracted from leaves dried in an oven at 50 °C and room temperature.

**Experimental approach:**

The drying experiments were initially carried out by oven drying and drying at room temperature until 10 % of moisture was achieved. Several essential oils were then extracted from the oven-dried samples for a preliminary study, followed by optimisation of the essential oil yield. The Box-Behnken design with response surface methodology was used to optimise the process and effectively analyse the influence and interaction of the factors on the essential oil yield. The chemical composition and functional groups of the essential oil from both samples were then analysed.

**Results and conclusions:**

The micrographs of the oven-dried leaves showed damage to the glandular trichomes. The analysis of variance showed significant linear, quadratic and interaction effects of particle size and time, as well as of microwave power and time on essential oil yield. The optimal conditions were: 30 min irradiation time, 550 W microwave power and a particle size of 0.5–0.7 mm, resulting in essential oil yields of (0.49±0.01) % from the oven-dried leaves and (0.40±0.01) % from the room-dried leaves, which accounted for (85.1±0.8) and (87.2±0.7) % of the oils, respectively, with linalool being the predominant component. Fourier-transform infrared spectra showed the presence of hydroxyl functional groups, confirming the chemical composition results.

**Novelty and scientific contribution:**

The results showed the effect of drying methods and microwave-assisted process parameters on the quantity of the produced essential oils. The essential oil yield obtained by the innovative microwave-assisted hydrodistillation results in an efficient process and product quality that can be a very useful ingredient for the food and pharmaceutical industry.

## INTRODUCTION

Medicinal and aromatic plants (MAPs) indeed occupy an important position in contemporary society, especially due to their versatile applications in various industries such as the pharmaceutical industry, food industry, cosmetics and personal care (*1*). These plants produce a rich blend of volatile compounds, known as essential oils, through their secondary metabolism (*2*). *Lippia adoensis* variety koseret is an indigenous Ethiopian culinary and aromatic herb belonging to the genus *Lippia*. There are five species within this genus, with *Lippia adoensis* being particularly well known for its use as a spice and in traditional medicine (*3*). Two different varieties of the plant are known, namely the wild variety (variety *adoensis*) and cultivated variety (variety koseret). The leaves have antimicrobial properties and are used for flavouring food and as a preservative (*4*, *5*). They are also used in traditional medicine to treat various skin diseases such as eczema and superficial fungal infections (*6*). The dried leaves, on the other hand, are powdered with barley and taken to treat gastrointestinal complaints (*7*).

The study of medicinal plants begins with pre-extraction and extraction techniques, which are crucial steps in isolating biologically active components from the plants (*8*). Various studies have shown significant variations in the yield and chemical composition of essential oils, which can be attributed to a number of interacting factors. These include ecological origin, environmental stress (such as soil type, humidity, mechanical damage, *etc*.), drying methods and extraction techniques (*9*). Drying is a widely used method for preserving medicinal and aromatic plants, as it extends shelf life and facilitates processing and transport. Numerous methods of drying herbs are known, including sun drying, drying at room temperature, freeze drying and oven drying. Oven drying at a temperature of 35–50 °C is the most common method used in laboratory-scale herb drying studies. It is often recommended for preserving heat-sensitive compounds in dried products (*10*). The choice of drying method can have a significant impact on the quality and quantity of essential oils in aromatic species (*11*). Therefore, when selecting the most suitable drying method for plant material, the moisture content should be reduced to 10–12 % to ensure optimal preservation of medicinal plants (*12*).

In recent years, new extraction methods such as microwave-assisted extraction, supercritical fluid extraction and pressurized solvent extraction have attracted considerable research attention (*13*). In contrast to traditional solvent extraction, microwave-assisted hydrodistillation (MAHD) consists in rapid microwave heating with almost no heat loss to the environment, as it occurs in a closed system. This efficiency allows the extraction process to be completed within minutes (*14*). Microwave heating relies on two main phenomena: ionic conduction and dipole rotation (*15*), which interact directly with polar materials or solvents. When applied to plant cells, microwave energy heats polar molecules, creating pressure that breaks down the cell walls, and thus increases extraction yield by releasing cell components into the solvent (*16*). Several factors including extraction time, microwave power and plant size affect the yield of microwave-assisted extraction. Problems such as insufficient stirring of the solvent and excessive swelling of the plant material can reduce the yield of the MAHD when using a lower solid-to-liquid ratio (*17*). To avoid this, a sufficient solvent volume must be ensured for complete immersion of the sample. Identifying optimal conditions therefore requires the use of statistical optimisation methods. Among the experimental designs used, the Box-Behnken design with the response surface methodology has proven much more effective than the classical approaches in its ability to analyse the effects of various factors and their interactions on the desired outcome (*18*).

Previous research has focused on the conventional method of extraction of essential oils from *Lippia adoensis* varieties koseret and *adoensis*. Mikre *et al*. (*19*) reported a yield of 0.19 % of hydrodistilled essential oil from the cultivated variety of *Lippia adoensis*, with linalool identified as the main component. Therefore, the aim of this study is to investigate the influence of irradiation time, microwave power and particle size on the yield of essential oils extracted from both oven-dried and room-dried leaves of *Lippia adoensis* variety koseret using microwave-assisted hydrodistillation. Several extracted essential oils were optimised using the Box-Behnken design and the response surface methodology.

## MATERIALS AND METHODS

### Sample collection and drying time

A mass of 10 kg of fresh leaves of *Lippia adoensis* var. koseret were harvested and collected at the Wondogenet Agricultural Research Center experimental field, which is roughly 267 km south of Addis Ababa, Ethiopia, situated at an altitude ranging between 1760 and 1920 m above sea level with mean annual rainfall of 1372 mm and temperature of 19 °C (*20*). The harvested leaves were immediately cleaned and rinsed. To determine the initial moisture content of the leaves, 5 g of fresh samples were measured using an analytical mass balance PX223KR/E (OHAUS Corporation, Parsippany, NJ, USA) and oven-dried at 105 °C until a constant mass was achieved (*21*).

For oven drying, the prepared samples were dried in a double-door oven UNE 600 (Memmert GmbH, Schwabach, Germany) with a capacity of 256 L at 50 °C until moisture content was 10 % and the time was then recorded.

For drying at room temperature, the prepared samples were dried at room temperature until the moisture content was 10 %. The average temperature and relative humidity of the room, measured using a thermometer/hygrometer (ANENG, Guangdong Jinyuanquan Electronic Technology Co., Ltd, Puning, PR China), were 25 °C and 51 %, respectively.

After the herbal materials were dried, the samples were further crushed and grounded using a coffee grinder HC-150 (Hario, Tokyo, Japan) and separated into particle sizes of 0.1–0.3, 0.3–0.5, 0.5–0.7 and 0.7–1.0 mm using Retsch AS 200 (Retsch GmbH, Haan, Germany) vibrating sieve shaker and stored in a sealed plastic bag in a dry place until extraction.

### Water activity determination

The water activity (*a*_w_) of the dried powdered samples was determined at 25 °C using a water activity measuring instrument (Novasina AG, Lachen, Switzerland), with a measuring range of 0.03 to 1.00 and a resolution of ±0.001.

### Field emission scanning electron microscope

The dried samples were mounted on SEM stubs using conductive carbon tape to ensure they were well grounded. The SEM equipment (Carl Zeiss AG, Oberkochen, Germany) was then started, along with initiating the vacuum system to achieve the high vacuum necessary for operation. The prepared samples were then loaded by placing the sample stub on the stage within the chamber and the chamber was closed. An appropriate accelerating voltage of 15 kV with a resolution of 1.0 nm and magnification of 25× was guaranteed. The working distance was adjusted and stigmation was corrected to focus the electron beam on the sample surface. Finally, images were taken using coarse focusing, fine focusing and image capture.

### Preliminary experiment with one factor

#### Effect of particle size on yield

To investigate how different particle sizes of 0.1–0.3, 0.3–0.5, 0.5–0.7 and 0.7–1.0 mm affect the yield of essential oil, the ground oven-dried samples were subjected to microwave-assisted hydrodistillation (MAHD) under constant extraction conditions of 30 min and 800 W. For the extraction, a microwave oven (SK MWO-20M5; SK Magic, Seoul, South Korea) with the following specifications was used: a maximum output power of 800 W and an operating frequency of 2450 MHz. The internal cavity of the microwave oven measured 460 mm (*b*)×360 mm (*h*)×280 mm (*l*). To connect the flask to the condenser, a hole was drilled in the top of the microwave oven, where a three-way adapter was attached. Inside the cavity of the microwave oven, a 2000-mL Pyrex round bottom flask containing 50 g of raw material and 500 mL of distilled water was placed. The extraction was then carried out and the extracted oil was measured and dried using anhydrous sodium sulfate (Daejung Chemicals & Metals, Siheung, South Korea). The yield of the essential oil was then determined using the following equation:


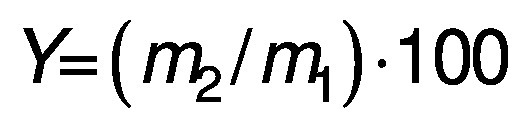
 /1/

where *Y* is the essential oil yield (%), *m*_2_ is the mass of essential oil obtained (g) and *m*_1_ is the mass of dried sample used (50 g).

#### Effect of microwave power on yield

Microwave power is another process parameter that influences the yield of essential oil. To investigate the effect of different microwave powers of 50, 300, 500 and 800 W on the essential oil yield of *Lippia adenosis*, 50 g of the oven-dried, ground sample with a constant particle size ranging from 0.7 to 1.0 mm were mixed with 500 mL of water and extracted by microwave-assisted hydrodistillation for 30 min. The collected essential oil was then dried with anhydrous sodium sulfate (Daejung Chemicals & Metals) and the yield was calculated using Eq. 1.

#### Effect of irradiation time on yield

Similarly, the effect of irradiation times of 10, 20, 30 and 40 min was investigated by extracting 50 g of oven-dried sample in 500 mL of distilled water while maintaining a constant microwave power of 800 W and particle sizes between 0.7 and 1.0 mm. The collected essential oil was then dried with anhydrous sodium sulfate (Daejung Chemicals & Metals) and the yield was calculated using Eq. 1.

### Response surface methodology with Box-Behnken design optimisation and model fitting

Based on the results of the preliminary one-factor experiment, three independent variables (factors) were selected for further optimisation of the essential oil yield of *Lippia adenosis* (dependent variable Y) from both room-dried and oven-dried leaves. These factors were: particle size (A), microwave power (B) and extraction time (C), each consisting of three levels with low, intermediate and high values of 0.3, 0.5 and 0.7 mm; 300, 550 and 800 W; and 20, 30 and 40 min, respectively.

The response surface methodology (RSM) with the Box-Behnken design (BBD) was further used to optimise the extraction process. The BBD generated a design matrix that outlined a total of 17 experimental runs with 5 central point replications for each oven and room-dried sample. The extraction experiment was then carried out based on the generated design matrix with a fixed mass of 50 g of raw material and volume of 500 mL of distilled water. The collected essential oil was then measured and the yield was calculated using Eq. 1.

To correlate the relationship between the essential oil yield (response variable) and the three independent variables, the Box-Behnken design was used to fit a second-order mathematical model, which can be expressed in the following equation:


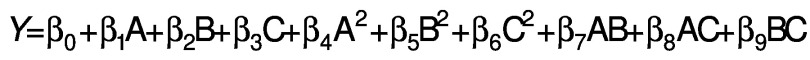
 /2/

where *Y* is the predicted essential oil yield, A (X_1_) is the particle size and B (X_2_) and C (X_3_) are the microwave power and irradiation time, respectively. The coefficient β_0_ is a constant, while β_1_…β_9_ are the regression coefficients for linear, quadratic and interactive terms, respectively, which are determined by regression analysis and represent the contributions of the corresponding variables and their interactions to the essential oil yield. The independent variables (A, B and C) were coded as low value (-1), central point (0) and high value (+1), respectively, using the following equation:


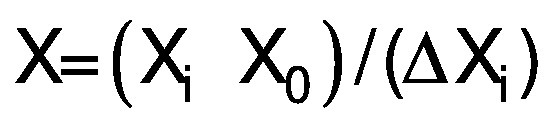
 /3/

where X is the coded value of X_i_, X_0_ is the value of the independent variable at the centre point, ΔX_i_ is the step change at i=1,2,3.

The regression coefficients were finally applied for model fitting analysis to generate response surfaces in three-dimensional plots and contour maps in two-dimensional plots which facilitated the visualisation of the interactive effects of the variables on the response variable. Finally, the validity of the predicted model for the essential oil yield was confirmed by analysing the model adequacy and considering the desirability of outcomes. The experiments were carried out in triplicate for each room-dried and oven-dried sample to confirm the predicted yields.

### Gas chromatography analysis

The chemical composition of essential oils from room-dried and oven-dried leaves was analysed by 7890B gas chromatography (GC), 5977B mass selective detector (MSD) and G4513A autosampler (Agilent Technologies, Santa Clara, CA, USA) with Agilent HP-5 column (60 m×250 μm×0.25 μm; Agilent Technologies). The mass spectrometer used an electron impact (EI) ion source and was adjusted for the analysis of parameters: electron energy, quadrupole temperature, source temperature and scan mass of 70 eV, 150 °C, 203 °C and 30 to 350 *m*/*z*, respectively. Helium served as the carrier gas at a flow rate of 1.2 mL/min. The inlet temperature was 250 °C. Samples of essential oils were first diluted with ethanol (99.9 %; Duksan, Ansan, South Korea) at a dilution ratio 1:4, resulting in a dilution factor of 5. The injection volume of the sample was 0.5 μL into a split/spiltless inlet unit with a 10:1 split ratio. The analysis began at an initial temperature of 50 °C, maintained for 5 min, followed by a heating rate of 2 °C/min until reaching a final temperature of 300 °C. The composition of essential oil was then identified by comparing its spectral fragmentation patterns from the Adams libraries and determined quantitatively using peak area percentage (*22*).

### Fourier transform infrared analysis

The functional groups in essential oils were determined using the Model Spectrum 3 Fourier transform infrared (FTIR) spectrometer equipped with an attenuated total reflectance (ATR) MIRacle Diamond Frontier 119424 (PerkinElmer Inc, Waltham, MA, USA). The samples were prepared for testing by first cleaning the automatic target recognition crystal with 99.9 % ethyl alcohol (Duksan). The instrument was then calibrated and the background was scanned according to the manufacturer’s specifications and validated against a standard polystyrene reference spectrum. Samples were analysed by applying a drop of the essential oil directly onto the crystal using a Pasteur pipette and ensuring full contact with the surface. The ATR press was then closed to ensure contact between the sample and the crystal. By scanning in transmission mode, the electromagnetic spectrum was recorded at room temperature over a wavenumber range of 560-4000 cm^-1^, with a resolution of 4 cm^-1^ and 16 scan accumulations. The most important absorption peaks were finally identified from the obtained spectrum and compared with the available literature.

### Statistical analysis

The yield of essential oils from the preliminary study was used for the statistical analysis. Differences between the group means were determined using Tukey’s HSD test for comparison (*23*). Design-Expert v. 13 software (*24*) was used for the optimisation study, analysis of variance (ANOVA), multi-regression analyses and significance tests with a significance level of p<0.05.

## RESULTS AND DISCUSSION

### Drying time of samples

The moisture content of the fresh sample was (79.3±1.0) %. A clear difference in drying time was observed between room-dried and oven-dried samples. As expected, the higher temperatures in oven drying significantly reduced the drying time. Room-dried leaves required the longest drying time (96 h), while oven drying required the shortest drying time (15 h), which means that room drying was 6.4 times longer than oven drying at 50 °C. Factors such as the quantity of plant material, its moisture content and environmental conditions like temperature and humidity (*25*) affect the substantial difference in drying time between the different methods.

### Water activity of samples

Water activity (*a*_w_) is an important factor that affects the stability and safety of stored products. At lower values, such as 0.6, the proliferation of potentially harmful microorganisms, including fungi, is inhibited (*26*). The water activity values of the leaves of *Lippia adoensis* var. koseret, obtained by oven and room drying, were found to be 0.42±0.01 and 0.48±0.01, respectively. These values indicate that both drying methods reduce the water activity of the samples and these low values are associated with the reduced moisture content in the samples. Consequently, the lower water activity in the dried samples suggests that they are well-preserved and less susceptible to microbial contamination during storage.

### Field emission scanning electron micrographs

**Fig. 1:**
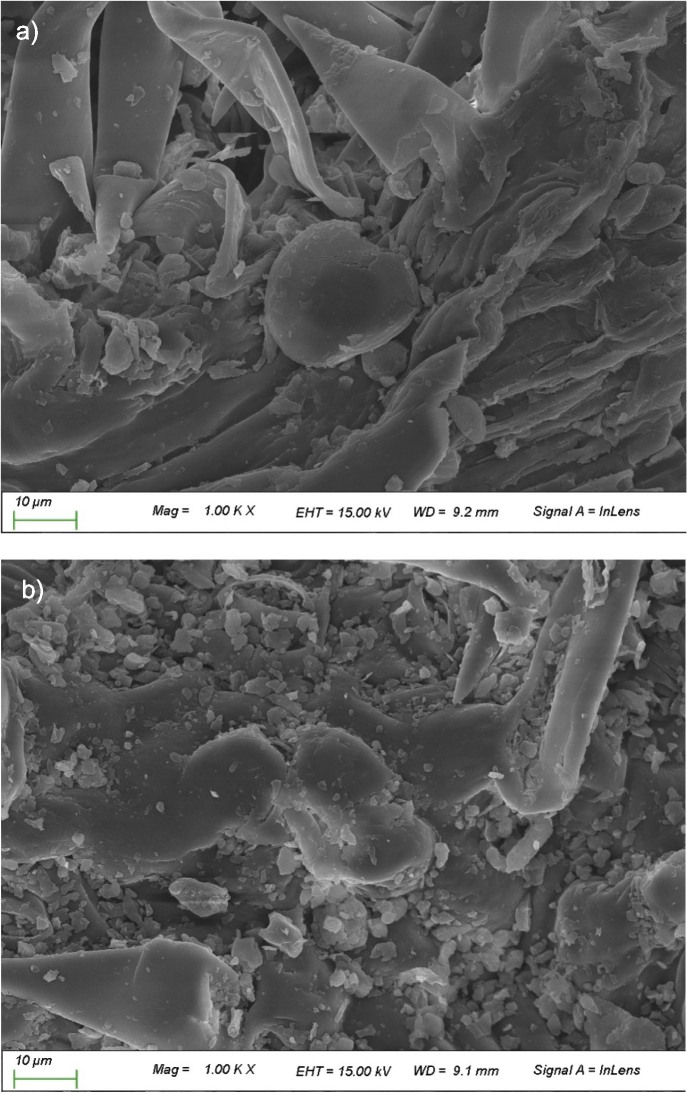
Scanning electron micrographs (SEM) of: a) leaves dried at room temperature, and b) oven-dried leaves at 50 °C

According to the SEM images of the glandular trichomes (Fig. 1), the cracking was more noticeable in the oven-dried samples than in the room-dried samples. The faster evaporation rate during oven drying than during room drying led to a faster water loss due to the applied heat, which caused the trichomes to dehydrate quickly and become damaged (*27*). In contrast, room drying better maintained the physical integrity of the trichomes due to the slower evaporation of water, which allows the trichomes to gradually adapt to the moisture loss. In a study by de Santana *et al.* (*28*), intact, deflated and ruptured glandular trichomes were observed at different drying temperatures in a forced air oven.

### Results of the preliminary experiment with one factor

#### Effect of particle size on the essential oil yield

**Fig. 2:**
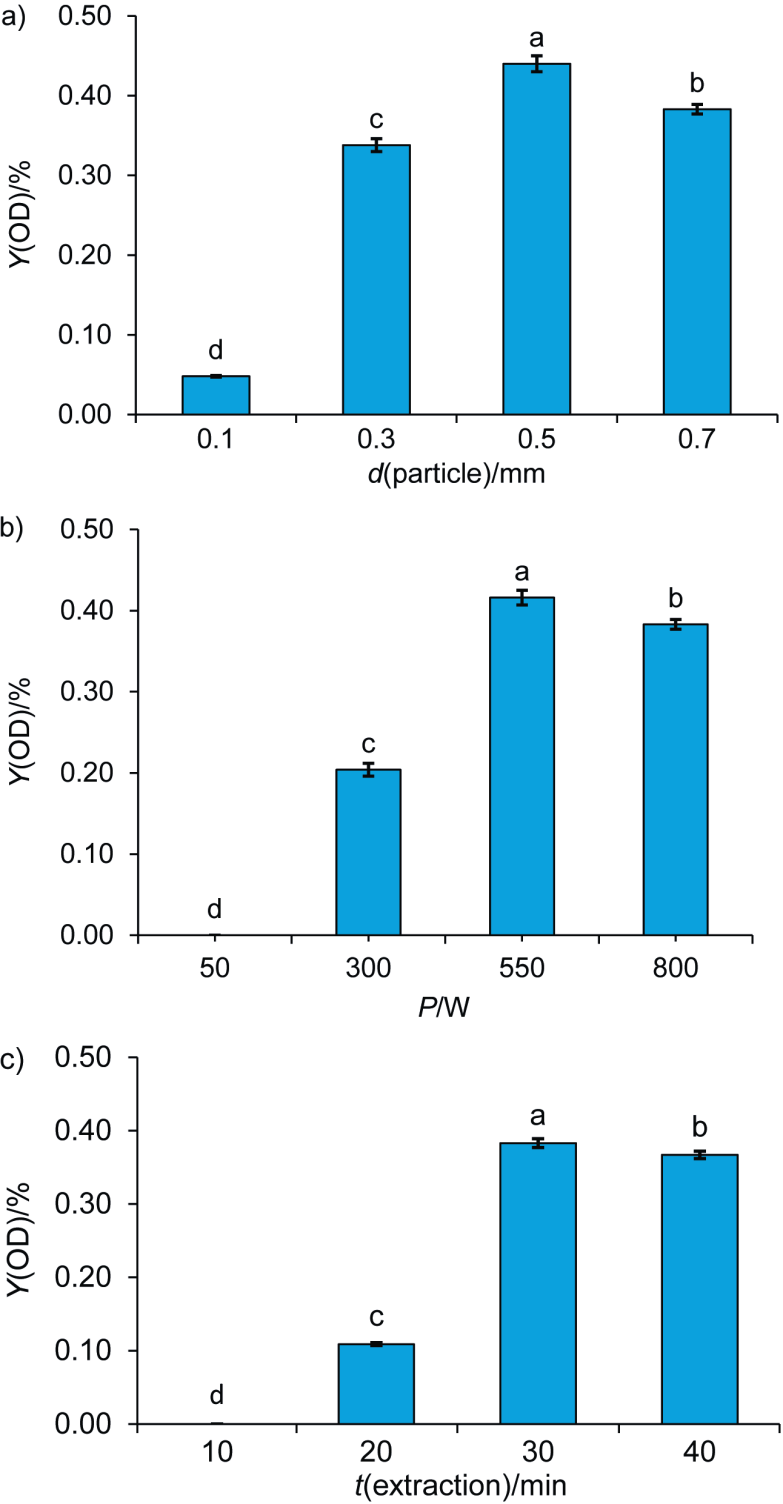
Single factor analysis of oven–dried (OD) samples: a) effect of particle size on essential oil yield, b) microwave power on oil yield, c) extraction time on essential oil yield. Different letters above the bars indicate statistically significant difference (p<0.05)

The results of the preliminary experiments with one factor are given in Fig. 2. At a fixed microwave power of 800 W and an irradiation time of 30 min, the effect of particle size (0.1–0.3, 0.3–0.5, 0.5–0.7 and 0.7–1 mm) on the yield of the essential oil showed an upward and downward effect, as shown in Fig. 2a and Table S1. Although the yield of most essential oils increases as the particle size decreases (*29*), the essential oil yield in this study significantly increased with the increase of particle size up to 0.5–0.7 mm. The lowest yield of the essential oil was obtained at a particle size of 0.1–0.3 mm and this smallest recovery compared to the others is due to the tendency of the extract to readily readsorb onto the surface of the *Lippia adoensis* powder. This phenomenon reduces the diffusion rate of the extracts, acting as a limiting factor in the extraction process (*30*). However, a decrease in the quantity of essential oil was again observed when the particle size increased beyond the range of 0.5–0.7 mm. This reduction can be attributed to the challenge the solvent faces in efficiently penetrating larger particles, thereby hindering the extraction process (*31*). Similar findings of the effects and higher extraction yield were achieved in the study by Tuan and Ilangantileket (*32*). The particle sizes from 0.3 to 1 mm were selected for the next optimisation.

#### Effect of microwave power on the essential oil yield

A significant increase in the yield of essential oil was observed when using power between 300 and 800 W while maintaining a constant particle size range from 0.7 to 1 mm and an extraction time of 30 min, as shown in Fig. 2b and Table S1. However, no recoveries were achieved at 50 W, which can be attributed to insufficient heat generation. This leads to a reduced dielectric heating effect, slower diffusion rates and less effective cell disruption (*33*). Conversely, a decrease in the yield of essential oil was observed at higher power (800 W) due to overheating and thermal degradation (*34*), as well as rapid solvent evaporation. A similar result was reported by Chen *et al.* (*35*). Based on these observations, lower, medium and higher microwave powers of 300, 550 and 800 W were selected for the next RSM optimisation.

#### Effect of irradiation time on the essential oil yield

Different extraction times from 10 to 40 min significantly affected the yield of essential oil, as shown in Fig. 2c and Table S1. Initially, an increase in the yield was observed until the extraction time reached 30 min, with no yield recorded at 10 min of extraction, which was carried out under constant conditions of the microwave power fixed at 800 W and particle sizes between 0.7 and 1.0 mm. The lack of essential oil yield for extraction time of 10 min was due to the inadequate absorption of microwave energy, which does not sufficiently increase the temperature of the water-plant mixture to effectively rupture the oil glands (*36*). The maximum yield was achieved at 30 min of exposure. However, extending the irradiation after 30 min resulted in a decrease in the oil yield, suggesting that this duration could represent a point beyond the equilibrium for extraction. The optimal conditions in this study are in close agreement with the 30 min irradiation time reported by Peng *et al*. (*37*) and this consistency in optimal irradiation time suggests that 30 min may be a critical parameter to maximise the yield of essential oil. Consequently, irradiation times ranging from 20 to 40 min were selected for further optimisation with RSM and BBD.

### Optimisation and model fitting with RSM and BBD

The yield of essential oil from the 17 experimental runs of the Box-Behnken design varied between 0.14 and 0.50 and between 0.11 and 0.41 % (by mass) for the oven-dried and room-dried samples, respectively, as shown in Table 1. Samples dried in the oven had a significantly higher essential oil content of (0.49±0.01) % than (0.40±0.01) % obtained from the room-dried samples under the optimal extraction conditions of power of 550 W, particle size of 0.5–0.7 mm and extraction time of 30 min. These optimal conditions suggested that the power of 550 W provided the right amount of heat to facilitate the release of the oil without degradation, the particle size of 0.5–0.7 mm allowed good solvent penetration and heat distribution, and the 30-minute extraction time ensured an effective and efficient process. On the other hand, the increase in essential oil content due to oven drying could be attributed to the response of the plant to stress, such as mechanical damage and heat (*38*), which triggers a series of biochemical reactions within the glandular trichomes and increases essential oil production. While several reports indicate that increased drying temperatures can damage the glandular trichomes of aromatic and medicinal plants (*39*), other studies have reported that higher drying temperatures can lead to an increase in essential oil yield in such plants (*40*). A similar increase in essential oil content was observed in *Dracocephalum kotschyi* Boiss plants with damaged glandular trichomes that were dried using a refractance window method as opposed to conventional shade drying (*41*). The leaves of *Lippia citriodora kunth* also showed a higher yield of essential oil when oven dried at 40 °C and vacuum dried at 60 °C than shade drying, freeze drying and vacuum drying at 40 °C (*42*).

**Table 1:** The response values of the Box-Behnken design

Run	*P*/W	*d*(particle)/mm	*t*/min	*Y*(OD)/%	*Y*(RD)/%
1	550	0.5	30	0.47±0.02	0.410±0.030
2	550	0.7	20	0.231±0.005	0.149±0.001
3	300	0.3	30	0.187±0.005	0.158±0.003
4	550	0.3	20	0.233±0.006	0.183±0.002
5	800	0.3	30	0.33±0.01	0.271±0.011
6	550	0.3	40	0.281±0.004	0.223±0.005
7	550	0.5	30	0.49±0.01	0.40±0.01
8	800	0.7	30	0.380±0.007	0.312±0.010
9	300	0.7	30	0.204±0.008	0.166±0.005
10	550	0.5	30	0.50±0.02	0.39±0.01
11	300	0.5	20	0.145±0.003	0.111±0.007
12	550	0.5	30	0.495±0.01	0.41±0.02
13	800	0.5	20	0.235±0.003	0.165±0.003
14	300	0.5	40	0.191±0.002	0.150±0.005
15	550	0.7	40	0.39±0.01	0.308±0.003
16	550	0.5	30	0.49±0.03	0.40±0.03
17	800	0.5	40	0.42±0.01	0.318±0.003

OD=oven drying, RD=drying at room temperature

Conversely, the experimental yields obtained with both drying methods were used to determine the coefficients of the quadratic model in Eq. 2. The regression coefficients of linear, quadratic and interaction terms of the model were determined and the obtained quadratic polynomial equation for the oven- and room-dried samples is shown in the following equations respectively:


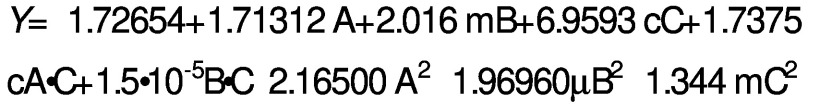
 /4/


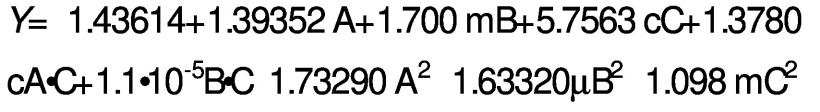
 /5/

Significance testing of the regression equations showed that the quadratic regression model for the essential oils obtained with both drying methods was found to be highly significant (p<0.0001), as shown by the ANOVA results (Table S2). The linear coefficients of microwave power and irradiation time as well as the quadratic terms were also highly significant. Moreover, the interaction coefficients between particle size and time, and between microwave power and time significantly affected the yield of essential oil obtained with both drying methods. In addition, the high predicted coefficients of determination R^2^ and the adjusted (0.9933 and 0.9866 for room drying and 0.9940 and 0.9879 for oven drying) indicate a strong correlation between the values predicted by the model and the actual values measured during the experiment, suggesting a high accuracy of the models (*43*). Additionally, the coefficient of variation, which quantifies the dispersion of data points around the mean, is low (4.42 % for oven-dried and 4.72 % for room-dried samples), suggesting that the results are consistently reliable across multiple experiments. The appropriate precision ratio, which also measures the signal-to-noise ratio, was 32.079 for room-dried and 33.645 for oven-dried samples, indicating the fitness of the developed models. The lack-of-fit F-values of 5.93 and 1.55 indicate that they are not significant compared to the pure error.

**Fig. 3:**
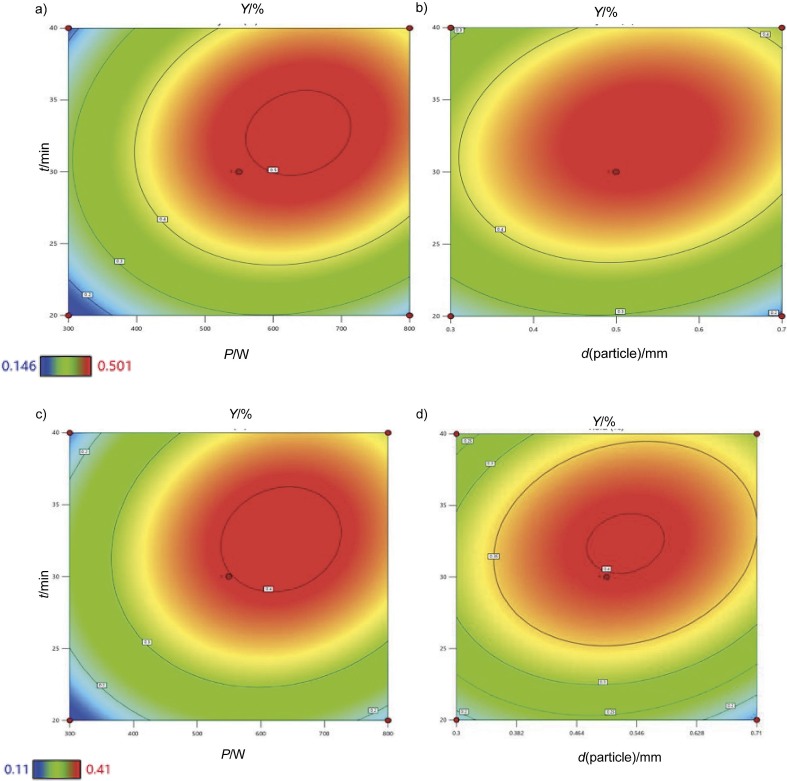
Contour plot of oven-dried samples: a) significant interaction between the power and time at p<0.05 and the fixed particle size of 0.5–0.7 mm, b) significant interaction between the particle size and time at p<0.05 and the fixed microwave power of 550 W, and room-dried samples: c) significant interaction between the power and time at p<0.05 and the fixed particle size of 0.5–0.7 mm and d) significant interaction between the particle size and time at p<0.05 and the fixed microwave power of 550 W. Red dots represent design points

**Fig. S1:**
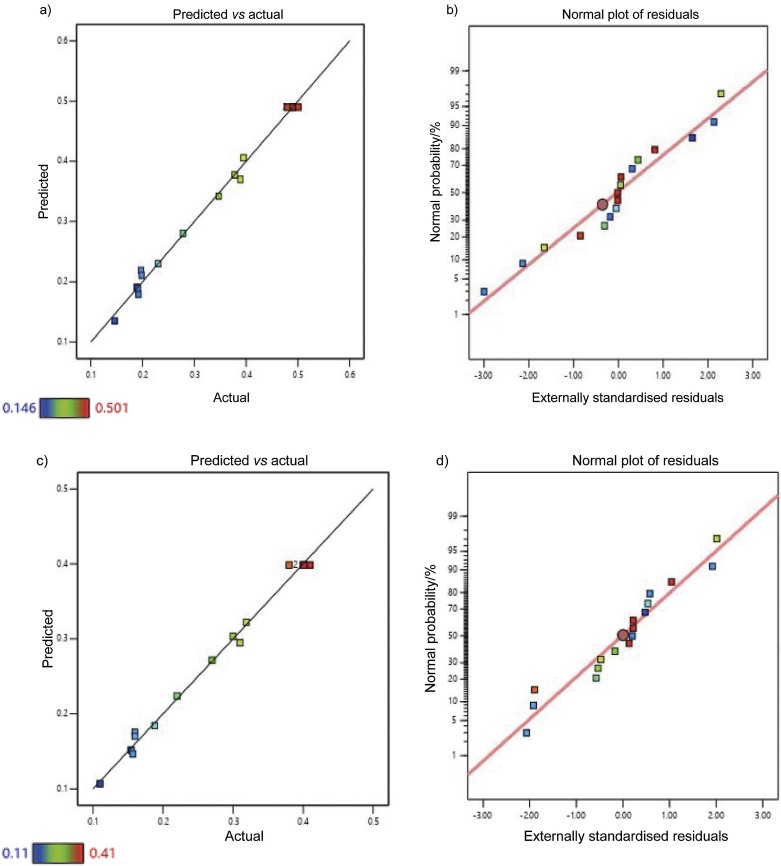
Model diagnostic plots of oven-dried samples: a) predicted *versus* actual value, b) normal probability plot of residuals, and room-dried samples: c) predicted *versus* actual value at the second standard condition (indicates that the model’s predictions are highly accurate), and d) normal probability plot of residuals

The visual representation of the interaction effects in contour plots and the model adequacy diagrams for both samples can be seen in Fig. 3 and Fig. S1. In Fig. 3a, a strong synergistic interaction between the microwave power and irradiation time was observed to have a significant effect on the essential oil yield, as shown by the steep gradient of the closely spaced contour lines and elliptical contour plots (*44*). A combination of 550 W microwave power, which provides the energy necessary for heating and the energy to break the cell walls, and a longer irradiation time of up to 30 min enables effective mass and heat transfer. This potentially accelerates the extraction process, in which has a great influence on essential oil yield (*45*).

Another significant interaction effect was observed between particle size and extraction time (Fig. 3b). This means that the surface area to volume ratio of the particle sizes, ranging from 0.5 to 0.7 mm, combined with a microwave irradiation time of 30 min is perfectly balanced to maximise the release of the essential oils, facilitated by enhanced heating and mass transfer (*46*). The other plot analysed is a plot of residuals, which indicates whether the residuals (differences between observed and predicted values) are normally distributed.

Similarly, the elliptical shape of the high-yield region of the room-dried samples in Fig. 3c suggests that deviations from the optimal combination of 550 watts and 30 min lead to a decrease in essential oil yield, which is relatively symmetrical in all directions away from the optimum. The elliptical shape of the zone with the highest yield is indicated in Fig. 3d by a bright red colour in contour plots centred around a combination of particle size values of 0.5–0.7 mm and extraction time of 30 min for the room-dried samples.

Analysis of the scatter plot of the predicted values *versus* the actual values of oven-dried samples (Fig. S1a) shows that the points generally follow the 45-degree line quite closely, indicating good predictive accuracy of the model for the samples.

As can be seen in Fig. S1b, the data points are close to the red reference line, but the tails of the distribution show some points that deviate from the line, which could indicate the presence of outliers or slight deviations from normality. However, these deviations do not appear to be extreme. In addition, the colour gradient representing different levels of yield does not show a clear pattern of the distribution of residuals. This suggests that the variance of the residuals does not depend on the yield level, which is good for the assumption of equal variance (*44*).

Comparatively, most of the data points of the room-dried sample are concentrated and nearly straight lines in the scatter plot of predicted *versus* actual values, indicating the stability of the model and its adherence to a normal distribution (Fig. S1c). However, the model predicts low yields with higher accuracy than high yields, as can be observed in the points moving further away from the line at higher yield values in the graph. The model was suitable for optimisation within the tested range.

The points in the plot of residuals in Fig. S1d follow the reference line closely, indicating that the residuals are normally distributed. In general, the model fitting analysis for both samples shows that the models are adequate and the dispersion of the experimental results is acceptable. Similarly, several studies using response surface methodology to optimise microwave-assisted extraction consistently emphasise that particle size, irradiation time and microwave power are statistically significant parameters that affect the yield of essential oil in different plants (*47*). On the other hand, the results of the model rechecking experiment in Table 2 showed that the model accurately predicts the responses under the given sets of conditions for both room-dried and oven-dried samples.

**Table 2:** Model validation

Factor			*Y*(OD)/%		*Y*(RD)/%	
*P*/W	*d*(particle)/mm	*t*/min	Experimental	Predicted	Experimental	Predicted
300	0.3–0.5	40	0.04	0.05	0.041	0.04
550	0.5–0.7	40	0.429	0.412	0.344	0.332
800	0.5–0.7	30	0.450	0.439	0.364	0.351

OD=oven drying, RD=drying at room temperature

Overall, the model and contour plot analysis showed that the best conditions for the extraction of essential oil yield were found with a particle size of 0.5–0.7 mm, a microwave power of 550 W and an extraction time of 30 min. These conditions resulted in a yield of (0.49±0.01) and (0.40±0.01) % for the oven-dried and room-dried samples, respectively.

Results of GC-MS analysis

**Fig. S2:**
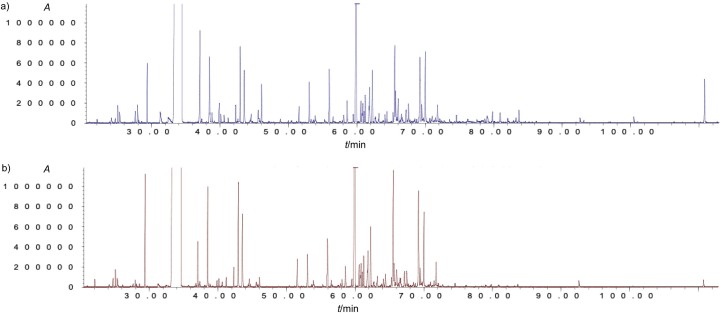
Results of gas chromatography analysis of *Lippia adoensis* essential oil extracted from: a) oven-dried samples and b) room-dried samples

In this study, a total of 49 and 46 compounds were identified by determining the peak area for oven-dried and room-dried samples, respectively (Table 3 and Fig. S2). The essential oils were found to contain oxygenated monoterpenes, sesquiterpenes and terpenoid alcohols. The main compound of the essential oils from oven- and room-dried samples was linalool, known for its various biological activities such as antimicrobial, anti-inflammatory, anticancer and antioxidant properties (*48*), accounting for (85.1±0.8) and (87.2±0.7) % of the total oil (100 %), respectively. Similarly, linalool has been reported to be a major component of *Lippia adoensis* variety koseret essential oil extracted using conventional hydrodistillation (*49*).

**Table 3:** GC-MS analysis of *Lippia adoensis* essential oil

*t*_R_/min	Compound	CAS number	*A*(OD)/%	*A*(RD)/%
27.712	Camphene	000079-92-5	(0.03±0.01)^b^	(0.06±0.01)^a^
29.794	Hexen-3-ol	004798-44-1	(0.06±0.01)^a^	(0.00±00.00)^b^
30.361	6-Methyl-6-hepten-2-one	000110-93-0	(0.03±0.01)^b^	(0.06±0.01)^a^
30.719	Myrcene	000123-35-3	(0.15±0.02)^a^	(0.08±0.02)^b^
30.994	3-Octanol	000589-98-0	(0.11±0.01)^a^	(0.08±0.01)^b^
33.277	*ortho*-Cymene	000527-84-4	(0.08±0.01)^a^	(0.03±0.01)^b^
33.604	Limonene	000138-86-3	(0.13±0.02)^a^	(0.05±0.02)^b^
33.807	1,8-Cineole	000470-82-6	(0.00±0.00)^b^	(0.03±0.01)^a^
34.214	*cis-*β*-*Ocimene	003338-55-4	(0.00±0.00)^b^	(0.02±0.01)^a^
35.016	*trans*-β-Ocimene	003779-61-1	(0.35±0.07)^b^	(0.8±0.1)^a^
36.919	*cis*-Linalool oxide (furanoid)	005989-33-3	(0.4±0.1)^a^	(0.06±0.02)^b^
40.081	Linalool	000078-70-6	(85.1±0.8)^b^	(87.2±0.7)^a^
42.726	Myrcenone	000539-70-8	(0.8±0.1)^a^	(0.23±0.09)^b^
42.977	*trans*-Tagetone	006752-80-3	(0.04±0.01)^b^	(0.06±0.01)^a^
44.114	Borneol	000507-70-0	(0.64±0.04)^b^	(0.82±0.04)^a^
44.495	*cis*-Linalool oxide	005989-33-3	(0.08±0.01)^a^	(0.02±0.01)^b^
45.777	α-Terpineol	000098-55-5	(0.07±0.01)^a^	(0.04±0.01)^b^
46.261	Myrtenal	000564-94-3	(0.08±0.01)^a^	(0.00±0.00)^b^
46.877	β-Cyclocitral	000432-25-7	(0.00±0.00)^b^	(0.06±0.01)^a^
47.936	valencene	0046-30-07-3	(0.24±0.09)^a^	(0.00±0.00)^b^
48.611	*cis*-Ocimenone	033746-71-3	(0.71±0.03)^b^	(0.96±0.05)^a^
49.206	Car-3-en-2-one	053585-45-8	(0.61±0.03)^b^	(0.73±0.04)^a^
50.083	Geraniol	000106-24-1	(0.50±0.01)^a^	(0.00±0.00)^b^
50.196	Linalyl acetate	000115-95-7	(0.08±0.01)^a^	(0.05±0.01)^b^
51.251	Geranial	000141-27-5	(0.18±0.02)^a^	(0.05±0.01)^b^
51.712	Perilla aldehyde	002111-75-3	(0.31±0.09)^a^	(0.06±0.01)^b^
57.197	Eugenol	000097-53-0	(0.14±0.05)^b^	(0.29±0.09)^a^
58.692	α-Copaene	003856-25-5	(0.42±0.06)^a^	(0.22±0.05)^b^
59.321	β-Bourbonene	005208-59-3	(0.04±0.01)^a^	(0.00±0.00)^b^
59.563	β-Copaene	018252-44-3	(0.16±0.06)^a^	(0.06±0.01)^b^
60.932	β-Guaiene	000088-84-6	(0.03±0.01)^a^	(0.00±0.00)^b^
61.617	*trans*-Caryophyllene	000087-44-5	(0.52±0.04)^a^	(0.33±0.05)^b^
63.542	*trans*-β-Farnesene	018794-84-8	(0.00±0.00)^b^	(0.02±0.01)^a^
63.743	α-Humulene	006753-98-6	(0.08±0.01)^a^	(0.05±0.01)^b^
64.223	Alloaomadendrene	025246-27-9	(0.28±0.06)^a^	(0.11±0.04)^b^
65.139	γ-Amorphene	006980-46-7	(0.17±0.06)^a^	(0.06±0.01)^b^
65.577	Germacrene D	023986-74-5	(2.5±0.5)^b^	(3.7±0.4)^a^
66.229	Cubebol	023445-02-5	(0.30±0.09)^a^	(0.12±0.04)^b^
66.411	Bicyclogermacrene	024703-35-3	(0.08±0.01)^a^	(0.00±0.00)^b^
66.506	α-Muurolene	031983-22-9	(0.17±0.06)^b^	(0.31±0.05)^a^
66.709	*trans*,*trans*-α-Farnesene	000502-61-4	(0.00±0.00)^b^	(0.13±0.05)^a^
66.884	β-Bisabolene	000495-61-4	(0.28±0.05)^a^	(0.16±0.05)^b^
67.513	*cis*-Muurol-5-en-4-β-ol	157374-46-4	(0.45±0.07)^a^	(0.31±0.05)^b^
67.914	δ-Cadinene	000483-76-1	(0.51±0.07)^a^	(0.35±0.06)^b^
68.872	*cis*-α-Bisabolene	070332-15-9	(0.17±0.05)^a^	(0.08±0.01)^b^
70.049	*cis*-Narolidol	000142-50-7	(0.13±0.05)^a^	(0.00±0.00)^b^
70.055	*trans*-Nerolidol	040716-66-3	(0.00±0.00)^b^	(0.13±0.07)^a^
70.940	α-Cadinene	082468-90-4	(0.12±0.05)^a^	(0.00±0.00)^b^
71.182	Germacrene D-4-ol	074841-87-5	(0.8±0.1)^b^	(1.03±0.06)^a^
71.329	Spathulenol	006750-60-3	(0.3±0.1)^a^	(0.12±0.04)^b^
71.691	Caryophyllene oxide	001139-30-6	(0.29±0.04)^a^	(0.12±0.04)^b^
72.173	Globulol	051371-47-2	(0.00±0.00)^b^	(0.06±0.01)^a^
72.268	*trans*-Veltonal	014398-40-4	(0.06±0.01)^b^	(0.09±0.01)^a^
72.845	Viridifloral	000552-02-3	(0.20±0.04)^a^	(0.11±0.03)^b^
75.126	γ-Cuprenene	004895-23-2	(0.14±0.05)^a^	(0.00±0.00)^b^
75.664	α-Cadinol	000481-34-5	(0.79±0.04)^a^	(0.47±0.01)^b^
87.690	Thujopsenal	000470-41-7	(0.04±0.01)^a^	(0.00±0.00)^b^
98.262	Isophytol	000505-32-8	(0.00±0.00)^b^	(0.06±0.01)^a^

Values are expressed as mean±S.D. (*N*=3). Mean values with different letters in superscript in the same row are significantly different (p<0.05). OD=oven drying, RD=drying at room temperature

The different drying methods led to considerable differences in the composition of the essential oils, with some components being absent from each method. Sesquiterpenes, like many other classes of terpenes, have benefits such as bactericidal properties (*50*) and anti-inflammatory effects (*51*). Components such as tujopsenal, γ-cuprenene, α-cadinene, *cis*-narolidol, bicyclogermacrene, valencene, β-guaiene, β-bourbonene, geraniol, myrtenal and hexene-3-ol were not found in the essential oils extracted from the room-dried leaves. Conversely, monoterpenes are also known for their many biological activities such as antimicrobial, hypotensive, antipruritic, antigerminative, antiplasmodial and anticandidal (*52*). In general, monoterpene and sesquiterpene hydrocarbons and their oxygenated derivatives are the main constituents of plant essential oils that have significant antimicrobial properties and effectively suppress microbial pathogens (*53*). These monoterpenes and certain sesquiterpenes such as 1,8-cineole, *cis*-β-ocimene, β-cyclocitral, *trans*-β-farnesene, *trans*,*trans*-α-farnesene, *trans*-nerolidol, globulol and isophytol were not detected in the essential oils extracted from oven-dried leaves. The variations in the concentration of the compounds during drying depend on numerous factors, such as the chosen drying method and the chemical structure of the compounds. These differences manifest themselves either as the loss or gain of oil constituents due to the formation of new compounds through processes such as esterification, oxidation, hydrolysis, glycoside and other biochemical transformations. This was reported by Agatonovic-Kustrin *et al.* (*54*), who also found a decrease in monoterpene content and an increase in sesquiterpenes content with increasing drying temperature.

### FTIR spectra

**Fig. 4:**
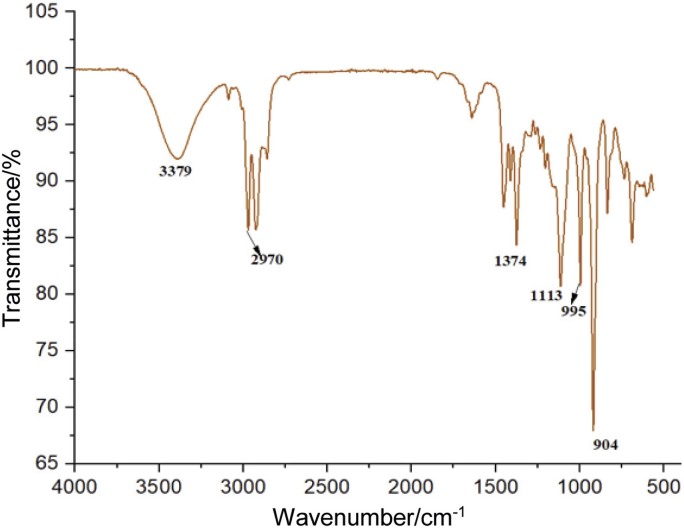
FTIR analysis of the samples

The Fourier transform infrared (FTIR) analysis of the spectra provides information about the chemical functional groups in *Lippia adoensis* essential oil in the frequency range of 560–4000 cm^-1^ (Fig. 4). The broad infrared band at 3379 cm^-1^ corresponds to the O-H (hydroxyl) group of alcohols, indicating the presence of alcohol in the essential oil. The bands at 2926 and 2970 cm^-1^ are attributed to non-uniform stretching vibrations of the C-H groups and they also correspond to the peak at 1374 cm^-1^. Another peak was observed at 1113 cm^-1^, which corresponds to C-O stretching vibrations. The strong peaks at 904 cm^-1^, relative to the others in the spectrum, are associated with (CH=CH_2_) stretching, indicating the presence of aromatic compounds, which are often characteristic of essential oils extracted from plants. Peaks around 1428 cm^-1^ are related to alkene C-H bending vibrations, indicating the presence of double-bonded hydrocarbons in the essential oil (*55*).

## CONCLUSIONS

This study highlights the importance and novelty of microwave-assisted hydrodistillation as an efficient method for the isolation of essential oils from the leaves of *Lippia adoensis* variety koseret. The use of response surface methodology with Box-Behnken design proved to be crucial in accurately predicting the optimal extraction parameters, ensuring an efficient process, higher yield and improved quality, which in turn contributes to cost reduction. An irradiation time of 30 min, a microwave power of 550 W and a particle size between 0.5 and 0.7 mm were identified as optimal conditions that ensure scalable results in industrial settings. Moreover, the results of GC-MS analysis showed that the quality of the oils was consistent, with a notable difference in the chemical composition, emphasising the effectiveness of the method and the potential for innovation in essential oil extraction. The extracted oils were rich in linalool, which is known for its flavour and therapeutic properties and therefore has a variety of applications. Therefore, this essential oil could be a promising product that can contribute to the development of effective food and pharmaceutical formulations.

## Appendix

**Table S1:** Single factor effect of particle size on essential oil yield

*d*(particle)/mm	*Y*(OD)/%	*P*/W	*Y*(OD)/%	*t/*min	*Y(*OD)/%
0.1–0.3	(0.0480.001)^d^	50	(0.000.00)^d^	10	(0.000.00)^d^
0.3–0.5	(0.330.01)^c^	300	(0.2040.008)^c^	20	(0.1090.002)^c^
0.5–0.7	(0.440.01)^a^	550	(0.4160.009)^a^	30	(0.3800.007)^a^
0.7–1.0	(0.3800.007)^b^	800	(0.3800.007)^b^	40	(0.3620.002)^b^

Values are expressed as mean±S.D. Different letters indicate statistically significant difference at p0.05. OD=oven drying

**Table S2:** Analysis of variance of the fitted models

Variable	Sum of squares^a^		DF	Mean square^b^		F-value		p-value		
	OD	RD		OD	RD	OD	RD	OD	RD
Model	0.2770	0.1845	8	0.0346	0.0231	164.61	147.86	0.0001	0.0001
A: particle size	0.0015	0.0011	1	0.0015	0.0011	7.32	6.93	0.0268	0.0301
B: power	0.0458	0.0286	1	0.0458	0.0286	217.48	183.09	0.0001	0.0001
C: time	0.0286	0.0183	1	0.0286	0.0183	135.76	117.54	0.0001	0.0001
AC	0.0048	0.0032	1	0.0048	0.0032	22.96	20.46	0.0014	0.0019
BC	0.0057	0.0031	1	0.0057	0.0031	27.09	20.10	0.0008	0.0020
A^2^	0.0316	0.0223	1	0.0316	0.0223	150.09	143.15	0.0001	0.0001
B^2^	0.0638	0.0439	1	0.0638	0.0439	303.28	281.23	0.0001	0.0001
C^2^	0.0760	0.0508	1	0.0760	0.0508	361.25	325.56	0.0001	0.0001
Residual	0.0017	0.0012	8	0.0002	0.0002				
Lack-of-fit	0.0014	0.0008	4	0.0004	0.0002	5.93	1.55	0.0564	0.3405
R^2^	0.9940	0.9933							
	0.9879	0.9866							
CV/%	4.42	4.72							
Adequacy precision	33.645	32.079							

^a^Sum of squares between average values and the overall mean, DF=degree of freedom, ^b^sum of squares divided by degree of freedom, F-value=test for comparing term variance with residual variance, p<0.05 was considered to be significant. OD=oven drying RD=drying at room temperature
